# AlloMAPS: allosteric mutation analysis and polymorphism of signaling database

**DOI:** 10.1093/nar/gky1028

**Published:** 2018-10-26

**Authors:** Zhen Wah Tan, Wei-Ven Tee, Enrico Guarnera, Lauren Booth, Igor N Berezovsky

**Affiliations:** 1 Bioinformatics Institute, Agency for Science, Technology and Research (A*STAR), 30 Biopolis Street, #07-01, Matrix, 138671 Singapore; 2 Department of Biological Sciences (DBS), National University of Singapore (NUS), 8 Medical Drive, 117579 Singapore; 3 Research School of Chemistry, The Australian National University, Canberra, ACT 2601, Australia

## Abstract

AlloMAPS database provides data on the causality and energetics of allosteric communication obtained with the structure-based statistical mechanical model of allostery (SBSMMA). The database contains data on allosteric signaling in three sets of proteins and protein chains: (i) 46 proteins with comprehensively annotated functional and allosteric sites; (ii) 1908 protein chains from PDBselect set of chains with low (<25%) sequence identity; (iii) 33 proteins with more than 50 known pathological SNPs in each molecule. In addition to energetics of allosteric signaling between known functional and regulatory sites, allosteric modulation caused by the binding to these sites, by SNPs, and by mutations designated by the user can be explored. Allosteric Signaling Maps (ASMs), which are produced via the exhaustive computational scanning for stabilizing and destabilizing mutations and for the modulation range caused by the sequence position are available for each protein/protein chain in the database. We propose to use this database for evaluating the effects of allosteric signaling in the search for latent regulatory sites and in the design of allosteric sites and effectors. The database is freely available at: http://allomaps.bii.a-star.edu.sg.

## INTRODUCTION

It is a common agreement nowadays that allosteric signaling is omnipresent (1) in regulation of activities of proteins (2) and molecular machines (3) with different structures and functions regardless of their sizes, oligomerization states and interactions with other molecules (4,5). Non-competitive and modulatory rather than on/off modes of action—both archetypal characteristics of allosteric signaling—opened an opportunity for design of allosteric drugs (6,7), which could help to avoid/reduce side effects, such as toxicity and receptor desensitization, typical for traditional orthosteric compounds (4,7,8). These advantages of allosteric effectors and growing number of success cases in the design of allosteric drugs (9–13) have fostered active research in the field of allostery. Additionally, clear indications of the allosteric effects of mutations (14,15) and their therapeutic potential (16), as well as recently reported abundance of deleterious mutations in allosteric sites (17) opens a new field of allosteric mutagenesis.

Freely available data and resources for investigating allosteric mechanisms (18–21) span from small datasets of so-called classical allosteric proteins with detailed description of experimentally determined sites and phenomenology of allosteric regulation to the benchmark sets and collections (22), and to the large literature-based lists of proteins, descriptions of allosteric modulatory actions, and allosteric networks derived from the analysis and crosslinking of different databases (23). Our goal here is two-fold: (i) to provide comprehensive data on allosteric causality and signaling obtained, for the first time, on the basis of a physics-based model and expressed in real energy units; (ii) to derive these data for both the selection of classical allosteric proteins and for almost two thousand PDBselect protein chains (24) with low sequence identity and diverse structures belonging to 411 CATH (25) topologies. Additionally, we analyzed 33 proteins with >50 SNPs, documenting allosteric effects of mutations and potential allosteric polymorphism.

Despite the wide diversity of biological functions involving allosteric regulation, the very molecular mechanism of allosteric communication is always determined by the protein structural dynamics, hence it can be formalized in the framework of a generic physical model. We have recently developed a structure-based statistical-mechanical model of allostery (SBSMMA), which allows one to quantify the free energy of allosteric modulation originated by the perturbations, such as ligand binding and mutations ((14,21,26), see also reference (7) in Tutorial). Reversibility of allosteric signaling, its potential for predicting allosteric sites, and for inducing required allosteric signaling from newly designated ones were also demonstrated (27). We have recently developed the AlloSigMA web server (21), which is an implementation of the SBSMMA (26). Contrary to earlier published SPACER server (18), which is based on more phenomenological concepts of binding leverage (2) and leverage coupling (3), the AlloSigMA allows one to evaluate real energetics of the allosteric signaling on the basis of SBSMMA (26). However, the computational cost in obtaining the exhaustive allosteric signalling maps (ASMs) in SBSMMA, especially in large proteins, made it impossible to perform this analysis on-line and motivated us to build a database comprising a large set of proteins with pre-calculated ASMs. The AlloMAPS database is a suite of interactive tools for exploratory analysis of causality and energetics of allosteric signaling. It quantifies direct and reverse allosteric signaling from/to allosteric sites and mutated residues, evaluating the modulatory effects of perturbations on the allosteric regulation, and allowing estimation of allosteric effects of non-native allosteric sites and mutations. The database can be used for exploring allosteric communication between known allosteric and functional sites, for the detection of potential latent regulatory sites, for using allosteric effects of mutations for direct modulation of protein activity, and for affecting the latter via tuning allosteric signaling from regulatory exosites.

## THEORETICAL BACKGROUND AND COMPUTATIONAL METHODS

**Figure 1.**
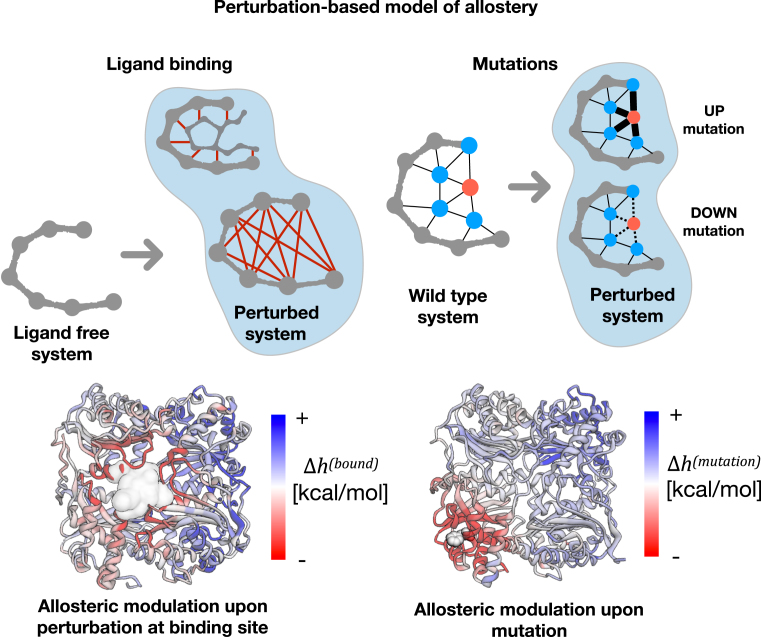
**Perturbation-based model of allostery used in calculations**. Top row: schemes of implementing the perturbations; ligand binding is mimicked by introducing strong interactions between residues of the binding site; mutations are modeled by increasing/decreasing force constants for interactions of mutated residue with its neighbors. Bottom row: Examples of the visualization of allosteric modulation caused by the ligand binding (left) and mutation (right).

The recently developed structure-based statistical mechanical model of allostery (SBSMMA, (26)) is used here for the calculation of allosteric free energy, or work exerted on the regulated sites and residues as a result of a perturbation such as ligand binding, mutations, or their combinations. SBSMMA is based on the harmonic model of a protein (Figure 1), where perturbations by the ligand binding are mimicked by increasing the interaction strength of contacts between residues of the binding site, and the effects of destabilizing/stabilizing mutations are modeled by weakening/strengthening interactions in the contact network of the mutated residue ((14,21), see also reference (7) in Tutorial). The model consists of sequential steps in which, first, configurational ensembles of the unperturbed (0) and perturbed (*P*) states are characterized by corresponding sets of orthonormal modes $e_\mu ^{(0)}$ and $e_\mu ^{(P)}$. Second, normal modes (in the normal mode analysis, the effective $C\alpha$ harmonic potential introduced in (28) is used to approximate the global dynamics of the proteins near equilibrium) are used for the calculation of an allosteric potential ${U_i}(\sigma ) = 1/2\sum\nolimits_\mu {{\varepsilon _{\mu ,i}}\sigma _\mu ^2}$, where ${\varepsilon _{\mu ,i}} = \sum\nolimits_j {{{| {{{\boldsymbol{e}}_{\mu ,i}}-{{\boldsymbol{e}}_{\mu ,j}}} |}^2}}$ are the parameters. The $\sigma = ( {{\sigma _1}, \ldots ,{\sigma _\mu }, \ldots } )$ is a vector of Gaussian variables with zero mean and variance $1/{\varepsilon _{\mu ,\ i}}$, each of which is associated with the corresponding sets of normal modes. The allosteric potential measures the total elastic work experienced by a residue as result of the change of displacement of its neighboring residues caused by a linear combination of normal modes, where the change of displacement of a residue is $\Delta {{\boldsymbol{r}}_i} = \sum\nolimits_\mu {{e_{\mu ,i}}{\sigma _\mu }}$. Third, by integrating the allosteric potential over possible configurations of the residue's neighbors, per-residue free energy difference between the free and bound states caused by the perturbation (*P*) is estimated

(1) \begin{equation*}\Delta g_i^{(P)} = \frac{1}{2}{k_B}T\sum\limits_\mu {\ln \frac{{\varepsilon _{\mu ,i}^{(P)}}}{{\varepsilon _{\mu ,i}^{(0)}}}} ,\end{equation*}

which depends exclusively on the parameters ${\varepsilon _{\mu ,i}}$ that characterize the unperturbed (0) and perturbed (*P*) protein conformational ensembles (see for details (14,21,26,27), also reference (7) in Tutorial).

The allosteric modulation (Figure 1), or background free allosteric effect, is evaluated as a deviation of the obtained free energy difference from its mean value over the protein chain:

(2) \begin{equation*}\Delta h_i^{(P)} = \Delta g_i^{(P)} - {\left\langle {\Delta g_i^{(P)}} \right\rangle _{Chain}}\end{equation*}

Allosteric modulation close to zero indicates that the response at the residue/site of interest is similar to the protein-average $\Delta g_i^{(P)}$ value, i.e. to the background effect on the whole protein. In order to monitor the effect of a perturbation on the functional sites of interest, the allosteric modulation per site is obtained as an average over all the residues belonging to the site:

(3) \begin{equation*}\Delta h_{SITE}^{(P)} = {\left\langle {\Delta h_i^{(P)}} \right\rangle _{i \in SITE}}\end{equation*}

In UP mutation $(m \uparrow )$, the strength of interactions in the contact network of the mutated residue is increased to simulate a substitution to a bulky one at residue *m*. Conversely, DOWN mutation $(m \downarrow )$ of a residue to small (Ala/Gly-like residue) is modeled by a decrease in the strength of interactions with its neighbors (Figure 1). The modulation range, a generic description of the allosteric effect of an amino acid substitution in a certain sequence position, is calculated as a difference between the responses caused by mutation from the smallest (Ala/Gly-like) to the bulkiest amino acids:

(4) \begin{equation*}\Delta h_i^{(m \downarrow \uparrow )} = \Delta h_i^{(m \uparrow )} - \Delta h_i^{(m \downarrow )}\end{equation*}

Large positive or negative $\Delta h_{SITE\ }^{(P)}$ and $\Delta h_i^{(P)}$ values correspond to an increase or decrease of work exerted on a residue/site by its neighbors, which may induce or prevent local conformational changes.

## DESCRIPTION OF THE DATABASE

The database provides access to massive data on allosteric signaling in about 2000 proteins and protein chains grouped in three sets. First, set of ‘Allosteric proteins’ includes 46 proteins with the knowledge on allosteric regulation well-documented on the basis of experimental works (27). This set of proteins can be used for exploring the causality and energetics of allosteric signaling between known functional and regulatory sites and for predicting possible locations of latent allosteric sites in these proteins (2). The ‘PDBselect chains’ set of 1908 protein chains contains representative structures with low sequence identity (less than 25%), allowing the user to survey allosteric signaling in a wide diversity of structures presented in the Protein Data Bank (29). Finally, the ‘Allosteric polymorphism’ set includes 33 proteins with multiple (more than 50 in each protein) known pathology-related SNPs, allowing the study of potential allosteric mechanisms in modulation or disruption of protein activity by mutations.

Given the list of available and annotated binding sites, effects of the ligand binding to these sites and their combinations on all residues of the protein are pre-calculated and can be immediately accessed. Modulation of these effects originated by individual mutations can be evaluated for mutations of residues designated by the user. Allosteric signaling from the sites with bound ligands to other known binding sites in the protein can be observed, along with cooperativity effects upon sequential binding of ligands to subunits of the oligomer. Data on allosteric modulation of the sites as a result of individual mutations of all residues are also available, as well as data on the effects of individual mutations and their combinations on all residues in the protein. Sequence positions undergoing stronger/weaker allosteric modulation or originating stronger/weaker allosteric modulation can be determined. The strongly modulating residues can be considered as candidates for comprising new, *de novo* designed, allosteric sites in order to achieve required allosteric signalling. The ASMs are precalculated for the cases of modulation range, UP, and DOWN mutations, and they are presented along with the protein distance matrices for the efficient analysis of allosteric signaling from the perspective of protein's structural traits.

All the data obtained by the user in the working session can be downloaded for further processing, using the ‘Download data’ button in the protein information panel accessible at the top of any page (see Tutorial for details).

## MATERIALS AND METHODS

The AlloMAPS database is written in Python using the Flask framework (http://flask.pocoo.org/). Calculation of the allosteric free energy and allosteric signaling maps is implemented in Python (26,27). The interactive web interface is powered by the JavaScript libraries D3.js (http://d3js.org/) and jQuery (http://www.jquery.com/). Structure visualizations are powered by PV (http://dx.doi.org/10.5281/zenodo.20980), and allosteric signaling maps are visualized using Plotly.js (http://plot.ly).

The database consists of three sets of proteins accumulated according to the criteria below. *Allosteric proteins*. This set, total 46 proteins, contains classic allosteric enzymes from previous studies (21,26,27) complemented by proteins from the benchmarking collection of allosteric proteins ASBench (22) on the basis of the following requirements (see (27) for more details): (i) if allosteric effects involve change of the oligomerization state and protein-protein interactions, protein records lack information on functional sites or part of the structure, or other relevant information is missing, these proteins were omitted; (ii) operational definition of allosteric sites (27) was used for obtaining list of proteins with true regulatory exosites. *PDBselect set*. We use the PDBselect as of Nov 2017, which contains 4184 protein chains with <25% sequence identity. Selecting structures belonging to 13 ‘most popular’ architectures according to CATH annotation, solved only by X-ray crystallography, and with sizes >30 amino acid residues, we analyzed 1908 protein chains, which represent 411 topologies and 984 homologous superfamilies, respectively. *Allosteric polymorphism*. Proteins with at least one sequence variant that is reported to be implicated in a disease and with a 3D structure were obtained from the list of human entries with polymorphisms or mutations (https://www.uniprot.org/docs/humpvar) from Uniprot. From this list of proteins, 33 proteins with at least 50 SNPs that are linked to disease(s) were collected using the human polymorphisms and disease mutations index (https://www.uniprot.org/docs/humsavar). Both humpvar and humsavar lists are obtained as of September 2017. There is at least one ligand-binding site in 27 proteins in this list.

## STRUCTURE OF THE DATABASE AND WEBSITE NAVIGATION

**Figure 2.**
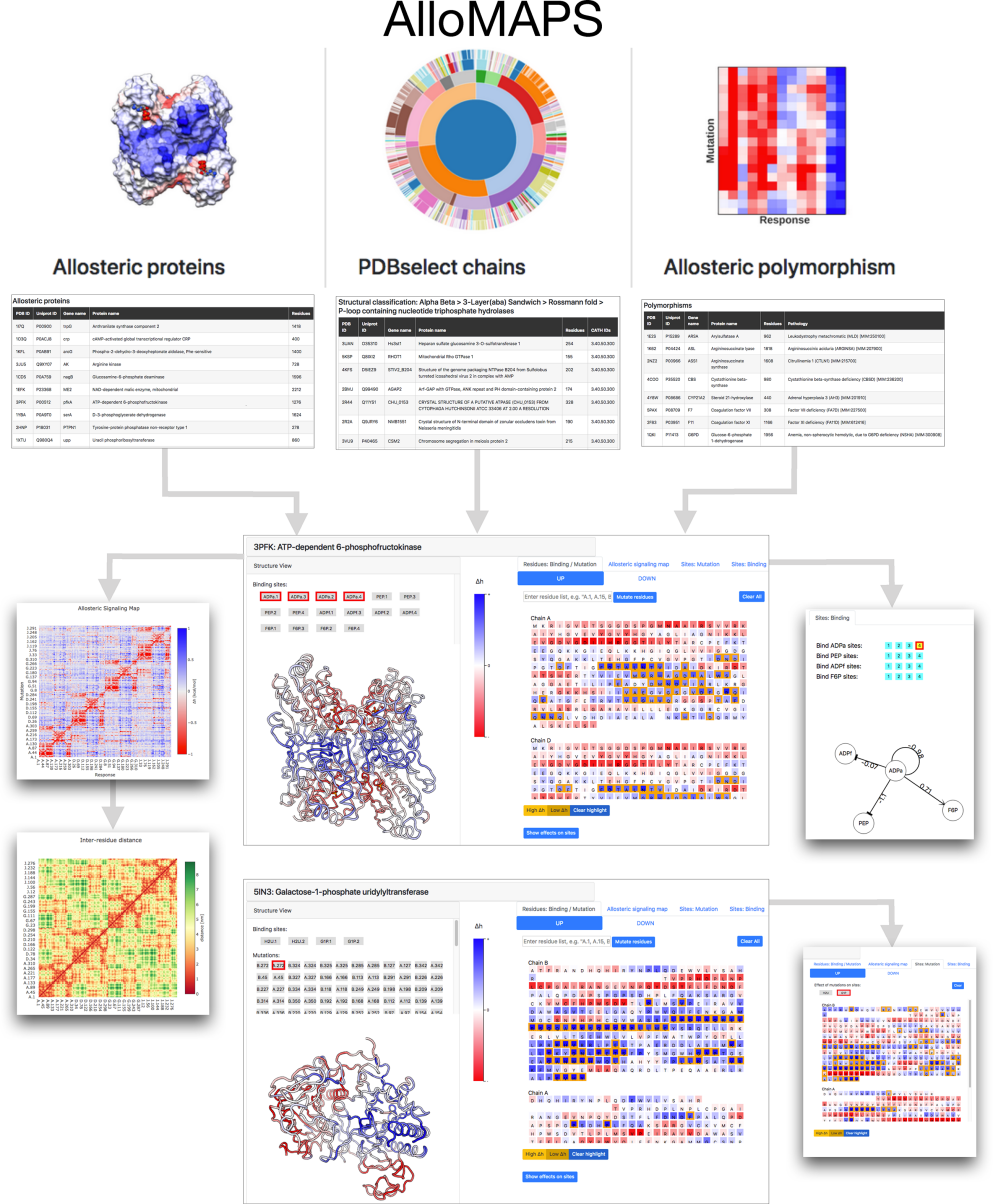
**Flowchart of the navigation through the AlloMAPS database**. Top row shows images of the homepage, which provides an access to three parts of the database: ‘Allosteric proteins’, ‘PDBselect chains’ and ‘Allosteric polymorphism’. List of proteins in these sets are exemplified below corresponding icons of the main page. Two big panels in the center show examples of typical main data-record pages. Small panels on the left show Allosteric Signaling Map (ASM, top) and corresponding distance map (bottom) for the protein. Top-right small panel is an example of visualization of the signaling from the site of interest (4xADPa—binding to sites in all subunits) to other binding sites (ADPf, F6P, PEP) in the protein. Bottom-right—effect of the SNP (A.272) on the selected binding site (G1P), with highlighting the sequence positions that induce strong modulation.

Figure 2 shows a flowchart navigating through the database. Starting from the homepage with three icons for ‘Allosteric proteins’, ‘PDBselect chains’ and ‘Allosteric polymorphism’ parts of the database, the user can see a list of proteins or protein chains (in case of ‘PDBselect chain’ option) in the corresponding part of the database. Choosing the required entry from the list, the user obtains an access to the main data-record page for the selected protein/protein chain. The page is divided into two parts: ‘Structure view’ on the left half, and ‘Sequence view’—right half. Below, the functional tabs and buttons are listed with a brief description of their role in the database.

‘Binding sites’ and ‘Mutations’ buttons located in the left structure view panel allow the user, by clicking on the button, to observe the corresponding site/residue in the structure and to obtain data on the role of these sites/mutations in allosteric modulation.

‘Residues: Binding/Mutations’ tab provides an access to the structure and sequence views in the data pages, allowing to analyze structure, allosteric effects of sites and mutations, effects on the sites (using button ‘Show effects on sites’ in the bottom of sequence view panel), and strongly/weakly modulated sequence positions as a result of the ligand binding and/or mutations (using ‘High Δ*h*’ and ‘Low Δ*h*’ buttons). Using tabs ‘UP’ or ‘DOWN’ user can analyze effects of stabilizing or destabilizing mutations in the sequence positions picked in the sequence view.

‘Allosteric signaling map’ tab provides access to the total scanning of single residues mutations. Tabs ‘UP’ or ‘DOWN’ visualize Allosteric Signaling Maps (ASMs) for stabilizing and destabilizing mutations, respectively. The tab ‘Modulation range’ shows ASM with a generic characteristic of the allosteric effect of the amino acid substitution in a sequence position—the difference between allosteric responses to mutation from the smallest (Ala/Gly-like) to bulkiest residue in the corresponding position. All ASMs are complemented by the matrices of the inter-residue distances in the protein/protein chain, which help to interpret the information contained in the ASM in relation to the structure and distances between allosterically communicating parts/residues of the protein.

‘Sites: Mutations’ tab allows one to observe effects of mutations on the binding sites described in the PDB file. By clicking on the button under the heading ‘Effect of mutations on sites’ user will obtain data on the allosteric signaling from all sequence positions to the corresponding site.

‘Sites: Binding’ tab provides a graph representation of the allosteric signaling from the analyzed site picked by the user to other binding sites listed in the PDB file. Cooperativity effects emerging upon sequential ligand binding to oligomer's subunits can also be observed by using buttons that designate the number of bound monomers.

## DATABASE USAGE

Figure 2 illustrates the database outputs, using 3pfk (ATP-dependent-6-phosphofructokinase) and 5in3 (galactose-1-phosphate uridylyltransferase) as examples. The 3pfk main data-record page (top center) shows results of the analysis of allosteric modulation in the PFK structure caused by the binding of ADPa activator to all four PFK subunits. A per-residue allosteric modulation caused by the 4xADPa binding is indicated by the color gradient in both the structure and sequence views. The ‘High *Δh*’ button shows residues under stronger than average positive modulation (highlighted by orange), *i.e*. residues likely undergoing conformational changes larger than the average of the protein/protein chains as a result of the perturbation caused by the 4xADPa binding. For proteins with multiple symmetry-related binding sites, the binding sites chosen by the user are perturbed concurrently, essentially mimicking the binding of multiple ligands to the corresponding sites in different subunits. We provide in the database the per-residue Δh values upon perturbation of any combination of symmetry-related binding sites. For example, user interested in the allosteric modulation upon perturbing two of the four available ADPa sites will have six combinations to choose from: ADPa.1 & 2, ADPa.1 & 3, ADPa.1 & 4, ADPa.2 & 3, ADPa.2 & 4 and ADPa.3 & 4. Two small panels on the left show the Allosteric Signaling Map (ASM) for the case of stabilizing UP mutations and the distance matrix for 3pfk. The ASM for UP mutations is provided by default via ‘Allosteric signaling map’ tab, ASMs for DOWN mutations and modulation range can be accessed through corresponding tabs. A small panel to the right from the main data-record page for 3pfk shows the allosteric signaling from 4xADPa liganded activator sites to other binding sites of the phosphofructokinase. The main-record panel in the bottom contains data for the ‘Allosteric polymorphism’ entry 5in3. The original record collected from different sources contains the information on both binding sites and SNPs (see buttons in the structure view on the left). The output shows an allosteric effect of one SNP (chain A, residue 272) indicated in the color gradient on sequence positions and structure representation. The strongly positively modulated positions are highlighted in the sequence view (orange) using the ‘High Δ*h*’ button. The small panel in the bottom right shows how UP mutations of different of sequence positions can affect the selected type of the site (here, G1P sites), with sequence position providing stronger than average positive modulation highlighted by orange in the sequence view. Effects of DOWN mutations can be observed by using the corresponding ‘DOWN’ tab. Given observed energetics of allosteric signaling from known SNPs and analyzing corresponding ASMs, the user can obtain a picture of allosteric polymorphism, where sequence positions other than ones with known SNPs can originate similar allosteric effects.

## CONCLUSION

AlloMAPS database provides unique opportunities for exploring allosteric regulation of protein activity, as it combines analysis of about 2000 proteins and protein chains representing wide diversity of sequences, structures, and functions, with a rigorous calculation of per-residue allosteric free energy on the basis of the recently developed structure-based statistical mechanical model of allostery (SBSMMA, (21,26,27), see also reference (7) in Tutorial). Causality and energetics of allosteric signaling analyzed for known allosteric sites and SNPs allows the user to obtain important estimates on the energetics of allosteric effects, which can be used in the search for latent allosteric sites and in the design of new allosteric ligands. Several instruments in the database are aimed at modeling direct allosteric signaling caused by the ligand binding and mutations, as well as at exploring and using the modulatory effects of sites and mutations on the energetics of other sites and sequence positions. Different combinations of binding and mutations can also be considered. Allosteric Signaling Maps (ASMs) obtained from the total scanning of mutations is the first exhaustive description of the allosteric signaling in per-residue resolution. In addition to the comprehensive description of stabilizing and destabilizing mutations, it provides a generic quantitative characteristic of the allosteric effect of a sequence position — ASMs of the modulation range. We expect that ASMs for stabilizing/destabilizing mutations and for modulation range will be used as a valuable source of information on the energetics of allosteric signaling in different design efforts. In particular, ASMs provide an opportunity to estimate modulatory effects of mutations, to combine signaling from several protein residues in order to achieve required strength of allosteric response, and even to estimate signaling from potential new sites built as a combination of considered residues. To conclude, we hope that AlloMAPS can become instrumental in targeting important tasks in protein engineering and design, such as prediction of latent regulatory exosites, evaluation of allosteric effects of mutations, modulation of protein activity and effects of the ligand binding via directed mutagenesis, and, finally, harmonized design of allosteric sites and effectors.
